# New point-of-care calcaneal ultrasound densitometer (Osteosys BeeTLE) compared to standard dual-energy X-ray absorptiometry (DXA)

**DOI:** 10.1038/s41598-024-56787-8

**Published:** 2024-03-22

**Authors:** Giovanni Adami, Maurizio Rossini, Davide Gatti, Paolo Serpi, Christian Fabrizio, Roberto Lovato

**Affiliations:** 1 Rheumatology Unit, Azienda Ospedaliera Universitaria Integrata di Verona, Pz Scuro 10, 37134 Verona, Italy; 2 Caresmed SRL, Milan, Italy; 3 Bone Specialist Unit, Ospedale Casa di Cura Villa Berica, Vicenza, Italy

**Keywords:** Quantitative ultrasound (QUS), Bone mineral density (BMD), Calcaneal ultrasound densitometer, Dual-energy X-ray absorptiometry (DXA), Rheumatology, Risk factors, Endocrine system and metabolic diseases

## Abstract

Dual-energy X-ray absorptiometry (DXA) represents the gold standard for measuring bone mineral density (BMD). However, its size and bulkiness limit its use in mass screening. Portable and easily accessible instruments are more suitable for this purpose. We conducted a study to assess the repeatability, sensitivity, accuracy, and validation of a new ultrasound densitometer for the calcaneus (OsteoSys BeeTLe) compared to standard DXA. BMD (g/cm^2^) was measured at the femoral and lumbar spine levels using DXA (Discovery Acclaim (Hologic, Waltham, MA, USA) or Lunar Prodigy (GE Healthcare, Madison, WI, USA) devices). Bone Quality Index (BQI, a dimensionless measure of bone quality derived from measures of SOS [Speed Of Sound] and BUA [broadband ultrasound attenuation]) was measured with OsteoSys BeeTLe. The Bland–Altman test and simple linear regression were used to evaluate the association between values measured with the two instruments. Additionally, the ability of the T-score calculated with BeeTLe to identify patients with previous osteoporotic fractures was tested using ROC curves. A total of 201 patients (94.5% females) with a mean age of 62.1 ± 10.2 were included in the study. The BeeTLe instrument showed a coefficient of variation (CV, in 75 repeated measurements) of 1.21%, which was not statistically different from the CV of DXA (1.20%). We found a significant association between BQI and BMD at the femoral neck (r2 = 0.500, p < 0.0001), total femur (r2 = 0.545, p < 0.0001), and lumbar spine (r2 = 0.455, p < 0.0001). T-scores bias were 0.215 (SD 0.876), 0.021 (SD 0.889) and 0.523 (SD 0.092), for femoral neck, total hip and lumbar spine respectively. AUC for discriminating fracture and non-fractured patients were not significantly different with OsteoSys BeeTLe and standard DXA. In this preliminary study, BeeTLe, a new point-of-care ultrasound densitometer, demonstrated good repeatability and performance similar to DXA. Therefore, its use can be proposed in screening for osteoporosis.

## Introduction

Osteoporosis is a widely diffused skeletal disorder characterized by reduced bone mass and deteriorated microarchitecture, leading to bone fragility and fractures^1^. Osteoporosis is a global health concern, particularly among the aging population, leading to substantial morbidity, mortality, and healthcare costs^2^. To mitigate the impact of osteoporosis, timely and accurate assessment of bone mineral density (BMD) is crucial. BMD serves as a fundamental metric for diagnosing osteoporosis, determining fracture risk, and guiding therapeutic interventions^3,4^. Dual-Energy X-ray Absorptiometry (DXA) has long been the gold standard for BMD measurement^5^. However, its limitations in terms of accessibility and portability necessitate the exploration of alternative technologies.

This study focuses on the diagnostic accuracy of a novel point-of-care calcaneal ultrasound densitometer, BeeTLe, in assessing BMD. The ability to provide reliable and convenient BMD assessments, particularly in mass screening scenarios, holds significant promise^6^. While DXA remains a cornerstone in osteoporosis diagnosis, innovative approaches like BeeTLe offer the potential to enhance accessibility and precision in bone health assessment. In this context, we aim to discuss the implications of our findings regarding the diagnostic accuracy of BeeTLe compared to DXA, emphasizing the broader implications for osteoporosis management and screening.

## Material and methods

### Study population

The study was a multicenter cross-sectional observational study on subjects fulfilling the following criteria:

Inclusion criteria: Caucasian ethnicity, aged 30–85 years, body mass index (BMI) < 40 kg/m^2^ and medical prescription for lumbar spine and femoral DXA investigation.

Exclusion criteria: patients with fewer than four lumbar vertebrae that could be evaluated on DXA due to presence of vertebral fractures or other artifacts, patients with bilateral hip fractures limiting standard DXA investigation, patients with bilateral ankle fracture or severe bilateral ankle osteoarthritis possibly limiting BeeTLe assessment.

Consecutive patients were recruited from two Italian centers from April 2023 and August 2023. The enrolled patients underwent a lumbar spine and femoral DXA investigation and calcaneus ultrasound densitometry (BeeTLe) approach, as detailed in the following paragraphs.

### DXA analysis

Anteroposterior DXA scans were performed using standard clinical procedures with either the Discovery Acclaim (Hologic, Waltham, MA, USA) or Lunar Prodigy (GE Healthcare, Madison, WI, USA) devices. For the total hip and femoral neck measurements, specific regions of interest were defined to capture BMD values. For the lumbar spine, a focused area spanning from L1 to L4 was scanned. These scans adhered to guidelines for positioning and patient alignment to ensure accurate and consistent measurements^7^.

The DXA scanner software automatically selected reference curves for T-score calculation based on patient characteristics, using databases such as NHANES III or proprietary manufacturer databases. All DXA scanners underwent daily quality control and regular maintenance throughout the study. BMD values measured by Lunar scanners were preliminarily converted in Hologic-equivalent values by applying specific conversion formulas derived from literature. For Hologic scanner, osteoporosis diagnosis criteria were as follows: lumbar spine was considered “osteoporotic” with a BMD ≤ 0.777 g/cm^2^ (T-score ≤  − 2.5), “normal” with BMD ≥ 0.932 g/cm^2^ (T-score ≥  − 1.0), and “osteopenic” for intermediate BMD values (− 2.5 < T-score <  − 1.0). The femoral neck was “osteoporotic” with BMD ≤ 0.577 g/cm^2^ (T-score ≤ −2.5), “normal” with BMD ≥ 0.733 g/cm^2^ (T-score ≥  − 1.0), and “osteopenic” for intermediate values.

The Lunar Prodigy scanner used different thresholds: lumbar spine “osteoporotic” with BMD ≤ 0.885 g/cm^2^ (T-score ≤  − 2.5), “normal” with BMD ≥ 1.054 g/cm^2^ (T-score ≥  − 1.0), and “osteopenic” for intermediate values. The femoral neck was “osteoporotic” with BMD ≤ 0.685 g/cm^2^ (T-score ≤  − 2.5), “normal” with BMD ≥ 0.854 g/cm^2^ (T-score ≥  − 1.0), and “osteopenic” for intermediate values.

### BeeTLe scans

OsteoSys BeeTLe is a portable wireless QUS (Quantitative Ultrasound) bone mineral densitometer characterized by its compact size, utmost lightness, and enough space for calcaneus measurement. It’s connectable to mobile phones or tablets by bluetooth and it consents easy and efficient measurement with data exporting by a dedicated App. It works with direct power supply or a built-in battery interchangeable with backup battery (≥ 5 V, 2A).

BeeTLe generates ultrasonic waves that pass through the heel of the foot and measure bone density. Through a proprietary algorithm, it calculates and displays the various bone density values of the patient. It measures SOS (Speed Of Sound) and BUA (broadband ultrasound attenuation), using ultrasounds with a central frequency of 0.5 MHz and calculates the BQI (Bone Quality Index) to show the degree of bone density for T-Score and Z-Score (T-scores and Z-scores calculated based on normal population of South Korea). It also performs measurements in Pediatric Mode if the patient’s age is under 18 years old.

### Repeatability

Repeatability was assessed on the first 15 patients included in the study (5 scans with BeeTLe, totaling 75 scans). Repeatability was expressed in terms of CV and LSC for a 95% confidence interval.

### Diagnostic accuracy

Diagnostic accuracy of BeeTLe was assessed by assuming DXA outputs as the gold standard reference and by determining sensitivity and specificity in the discrimination between “osteoporotic” and “non-osteoporotic” patients. Cohen’s Kappa (K) was used to evaluate the diagnostic accuracy of BeeTLe compared to DXA. Kappa < 0: no agreement; Kappa between 0.00 and 0.20: slight agreement; Kappa between 0.21 and 0.40: fair agreement; Kappa between 0.41 and 0.60: moderate agreement; Kappa between 0.61 and 0.80: substantial agreement; Kappa between 0.81 and 1.00: almost perfect agreement. To evaluate the relationship between values obtained with BeeTLe and DXA, linear regression analysis was conducted. This analysis included the calculation of the regression line's slope and the coefficient of determination (r2). The Bland–Altman test was employed to assess agreement and bias between BeeTLe and DXA measurements. This statistical analysis method revealed the degree of agreement and any potential systematic differences between the two measurement techniques. in discriminating between osteoporosis, osteopenia, and healthy bone.

All statistical analyses were performed using SPSS Version 26 (SPSS, Inc., Chicago, IL, USA) and GraphPad Prism version 9.5.1 (GraphPad Software, San Diego, CA, USA). This study was approved by the University of Verona ethic committee (prot. 1758CESC).

### Ethics approval and consent to participate

The study was conducted according to the protocol REUMABANK approved by our local Ethics Committee, in accordance with the 1964 Helsinki declaration and its later amendments or comparable ethical standards. Informed consent was collected for each participant.

## Results

**Figure 1:**
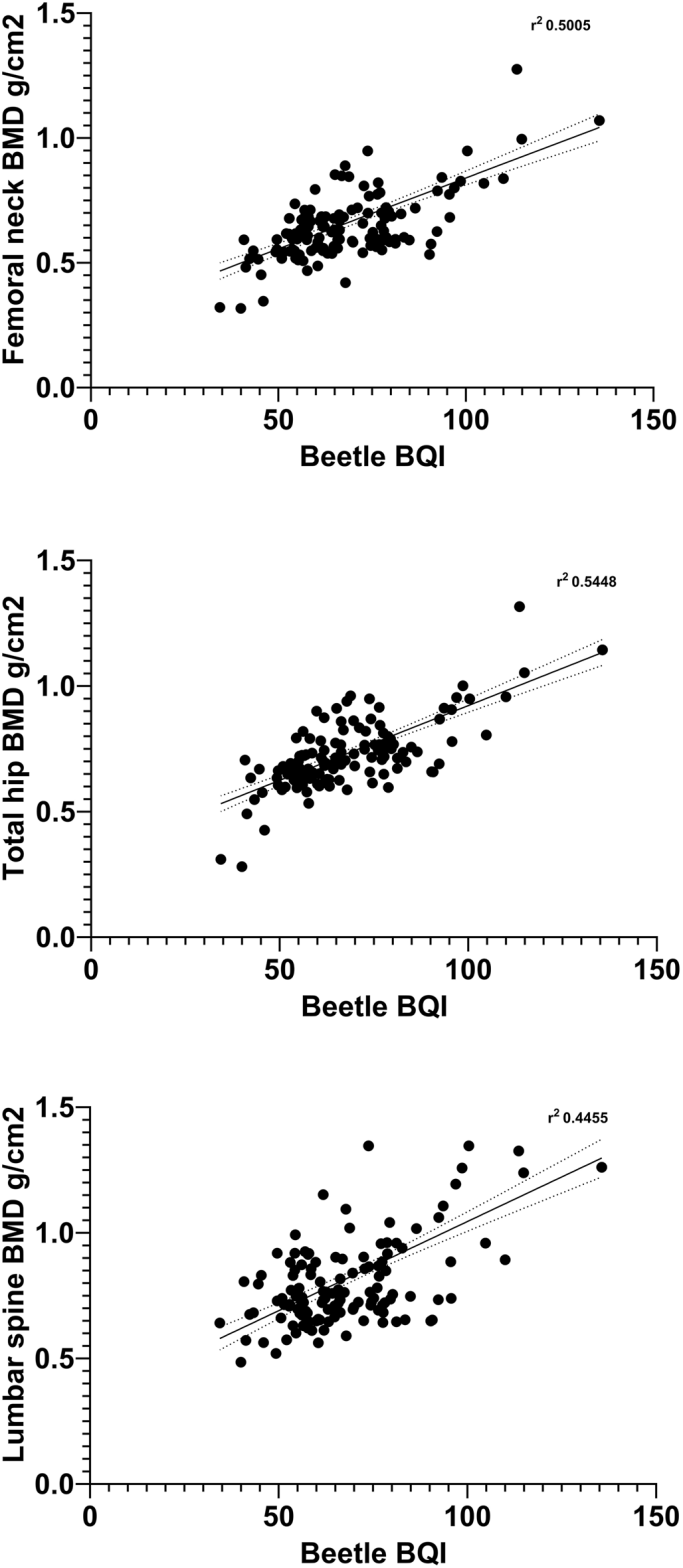
Regression between bone quality (BQI) index measure with Osteosys BeeTLE and bone mineral density (BMD) levels measured with dual-energy X-ray absorptiometry (DXA).

**Figure 2:**
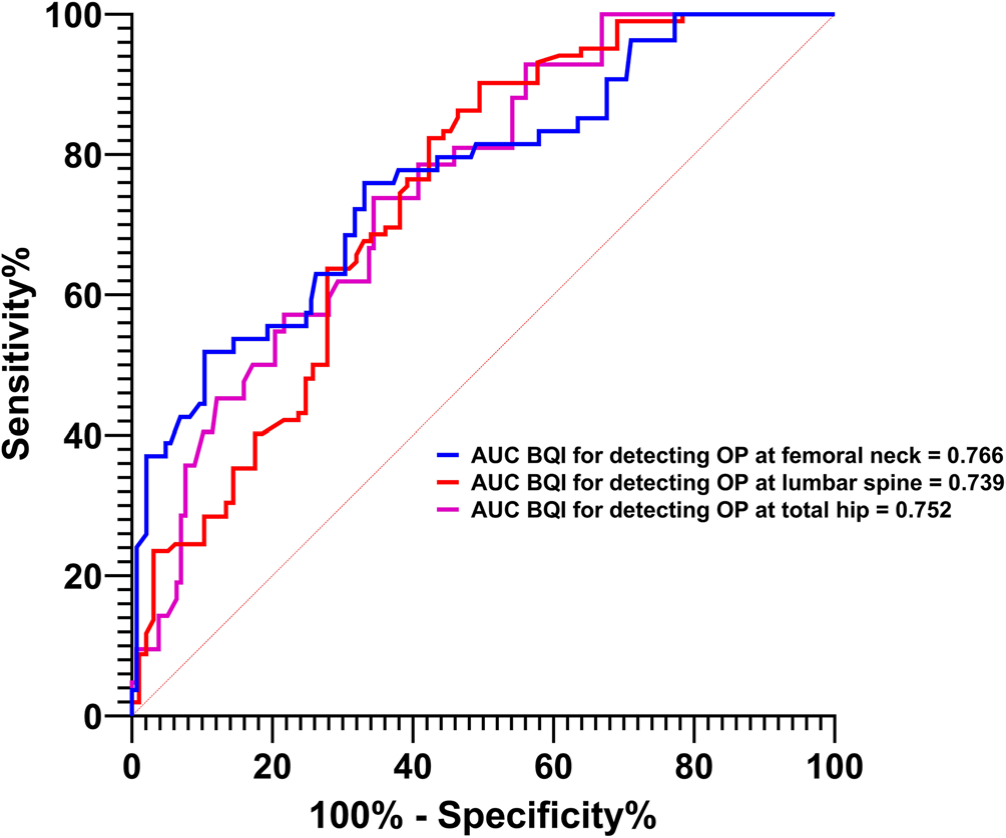
ROC diagnostic accuracy of Osteosys BeeTLE compared to standard DXA (osteoporosis defined as T-score <  − 2.5).

201 patients were included in the study. Table 1 shows the characteristics of the study sample. Figure 1 shows the regression lines and r2 for BMD levels measured and BQI. Figure 2 shows the ROC diagnostic accuracy of Osteosys BeeTLE. We found that the optimal threshold of BQI measured with Ostesys BeeTLE for discriminating osteoporotic patients (defined as T-score with standard DXA <  − 2.5) were: at femoral neck = 64.55—AUC 0.813; at total hip = 63.70—AUC 0.720; at lumbar spine = 78.25—AUC 0.786). Reference value for osteoporosis (T-score <  − 2.5) according to manufacturer is BQI = 58.8. Sensitivity and specificity of different BQI thresholds for discriminating osteoporotic patients are shown in Tables S1, S2 and S3. Supplementary Fig. S1 shows the distribution of T-score calculated with both DXA (lumbar spine, femoral neck and total hip) and Osteosys BeeTLE in the study population.

**Table 1:** Characteristics of the study population.

Characteristics	n = 201
Sex, female (%)	190 (95%)
Age, years (SD)	62.1 (10.2)
Menopause, n (% of 190 women)	182 (95%)
Weight, kg (SD)	59.3 (9.6)
Height, cm (SD)	163 (7.7)
Smoking history, n (%)	20 (10%)
Alcohol intake, n (%)	
None	164 (81.5%)
< 3 U/die	36 (18%)
> 3 U/die	1 (0.5%)
Family history of fragility fracture, n (%)	81 (40%)
Prior femoral fracture, n (%)	17 (8%)
Prior vertebral fracture, n (%)	62 (31%)
Thoracic vertebral	62 (100%)
Prior non-vertebral non-femoral fracture, n (%)	6 (3%)
Wrist	3 (50%)
Humerus	1 (17%)
Rib	2 (33%)
Comorbidities, n (%)	
Rheumatic musculoskeletal disease	25 (12%)
Diabetes	2 (1%)
Chronic kidney disease	19 (9%)
Prednisone ≥ 5 mg/day, n taking (%)	25 (12%)
Anti-osteoporosis medication, n (%)	
None (ever)	133 (66%)
Bisphosphonates	49 (24%)
< 1 year	15 (31%)
1–5 years	30 (61%)
> 5 years	4 (8%)
Denosumab	15(7%)
< 1 year	7 (47%)
1–5 years	5 (33%)
> 5 years	3 (20%)
Teriparatide	1 (1%)
< 6 months	1 (100%)
Romosozumab	3 (2%)
< 6 months	3 (100%)
Vitamin D supplements, n (%)	
0 U/day	11 (5.5%)
0–200 U/day	1 (0.5%)
200–400 U/day	14 (7%)
400–800 U/day	21 (10%)
800–1200 U/day	113 (56%)
> 1200 U/day	41 (20%)
DXA instrument	
Discovery acclaim hologic	122 (60%)
Lunar prodigy GE	79 (40%)
DXA femoral neck (SD)	
BMD g/cm^2^	0.673 (0.152)
T-score	− 2.0 (0.9)
Z-score	− 0.7 (0.8)
DXA total hip (SD)	
BMD g/cm^2^	0.747 (0.152)
T-score	− 1.8 (1.0)
Z-score	− 0.8 (0.9)
DXA lumbar spine (SD)	
BMD g/cm^2^	0.836 (0.201)
T-score	− 2.3 (1.7)
Z-score	− 0.8 (1.3)
TBS (SD), only in 63 subjects	1.319 (0.128)
Osteosys BeeTLE calcaneus (SD)	
BQI	70.7 (18.9)
T-score	− 1.8 (1.0)
Z-score	− 1.0 (0.8)

*SD* standard deviation, *DXA* dual-energy X-ray absorptiometry, *TBS* trabecular bone score, *BQI* bone quality index.

**Figure 3:**
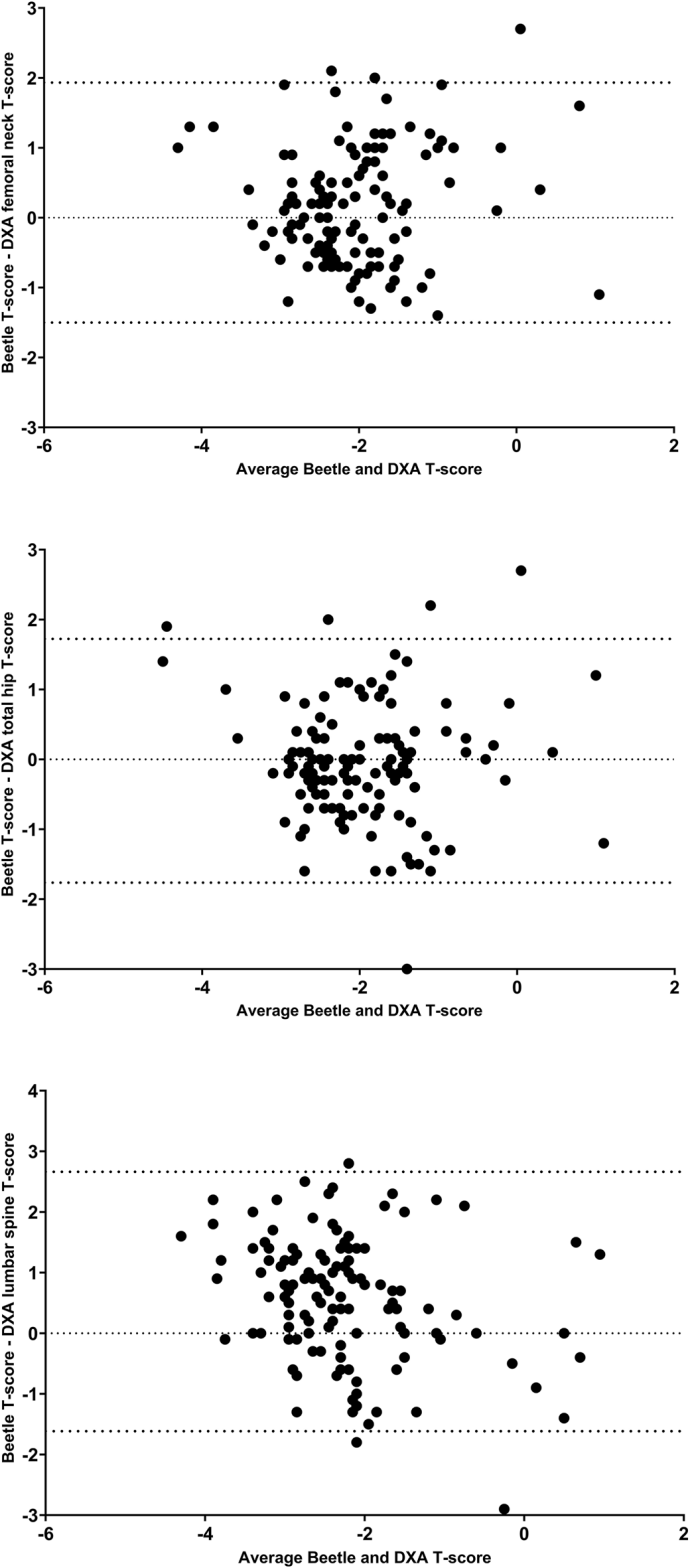
Bland Altman graph for Osteosys BeeTLE T-scores and dual-energy X-ray absorptiometry (DXA) T-scores.

Agreement between Osteosys BeeTLE and DXA was as follow: T-score Osteosys BeeTLE with total hip T-score Kappa = 0.326 SE of kappa = 0.058 95% confidence interval: from 0.213 to 0.439 Weighted Kappa = 0.427. T-score Osteosys BeeTLE with femoral neck T-score Kappa = 0.260 SE of kappa = 0.058 95% confidence interval: from 0.146 to 0.374. T-score Osteosys BeeTLE with lumbar spine T-score Kappa = 0.178 SE of kappa = 0.055 95% confidence interval: from 0.070 to 0.285. Figure 3 shows the Bland Altman graphs for Osteosys BeeTLE T-score and DXA T-score at various anatomical sites. For Osteosys BeeTLE compared to femoral neck, total hip and lumbar spine T-scores bias were 0.215 (SD of bias 0.876, 95% limits − 1.50 to 1.93), 0.021 (SD of bias 0.889, 95% limits − 1.76 to 0.72) and 0.523 (SD of bias 0.092, 95% limits − 1.62 to 0.66), respectively.

We conducted similar analyses on patients never exposed to anti-osteoporosis medications (n = 133). Figure S2 shows the ROC diagnostic accuracy of Osteosys BeeTLE in patients never exposed to anti-osteoporosis medications (98% without fracture, 100% without vertebral fracture). ROC curves were not significantly different from the overall sample. In this subgroup the agreement was as follow: T-score Osteosys BeeTLE with total hip T-score Kappa = 0.347 SE of kappa = 0.061 95% confidence interval: from 0.216 to 0.456 Weighted Kappa = 0.424. T-score Osteosys BeeTLE with femoral neck T-score Kappa = 0.252 SE of kappa = 0.059 95% confidence interval: from 0.145 to 0.383. T-score Osteosys BeeTLE with lumbar spine T-score Kappa = 0.169 SE of kappa = 0.054 95% confidence interval: from 0.075 to 0.279. Bland Altman graphs are shown in Fig. S3.

**Figure 4:**
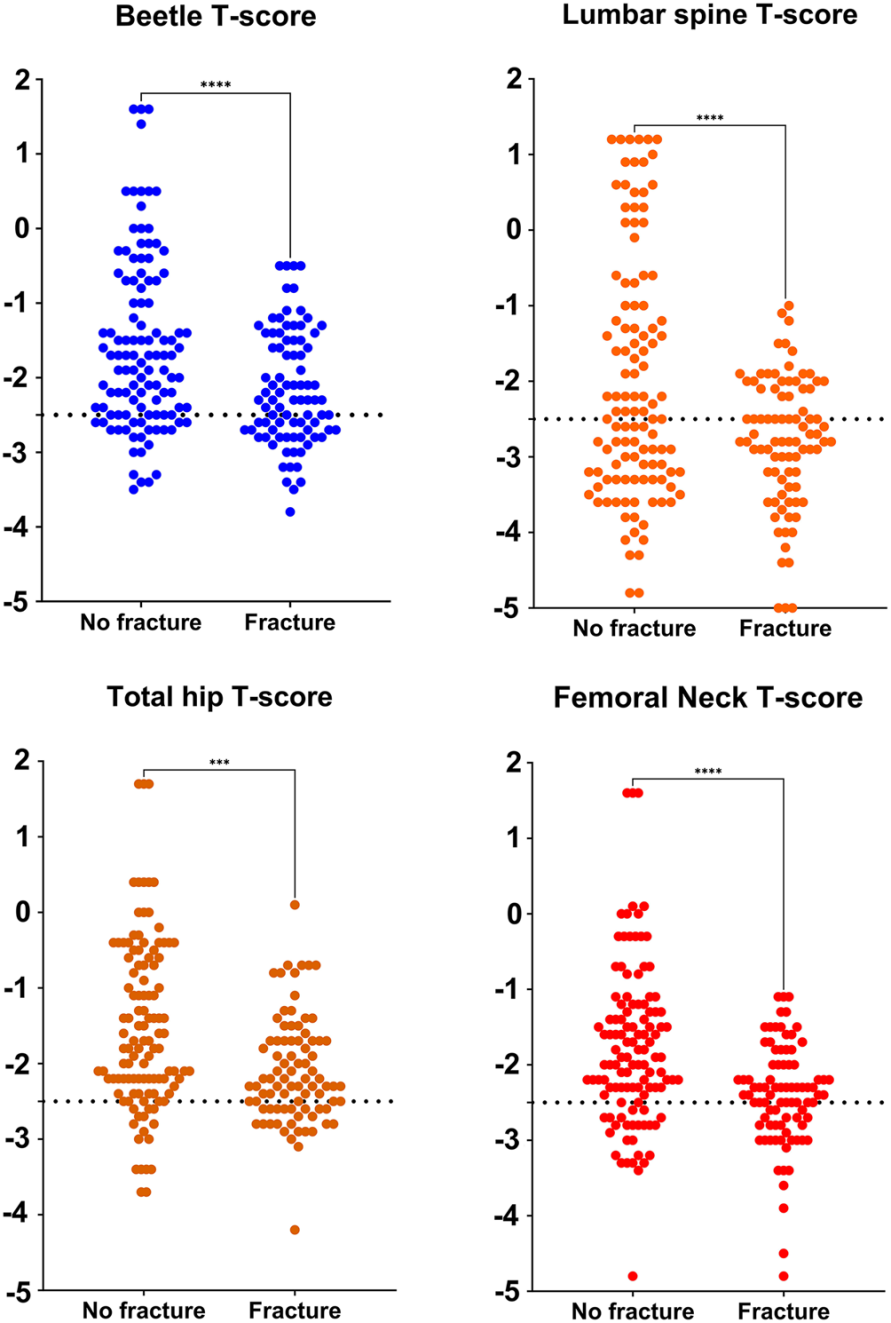
Osteosys BeeTLE and dual-energy X-ray absorptiometry (DXA) T-score levels in patients with or without prevalent fragility fracture.

**Figure 5:**
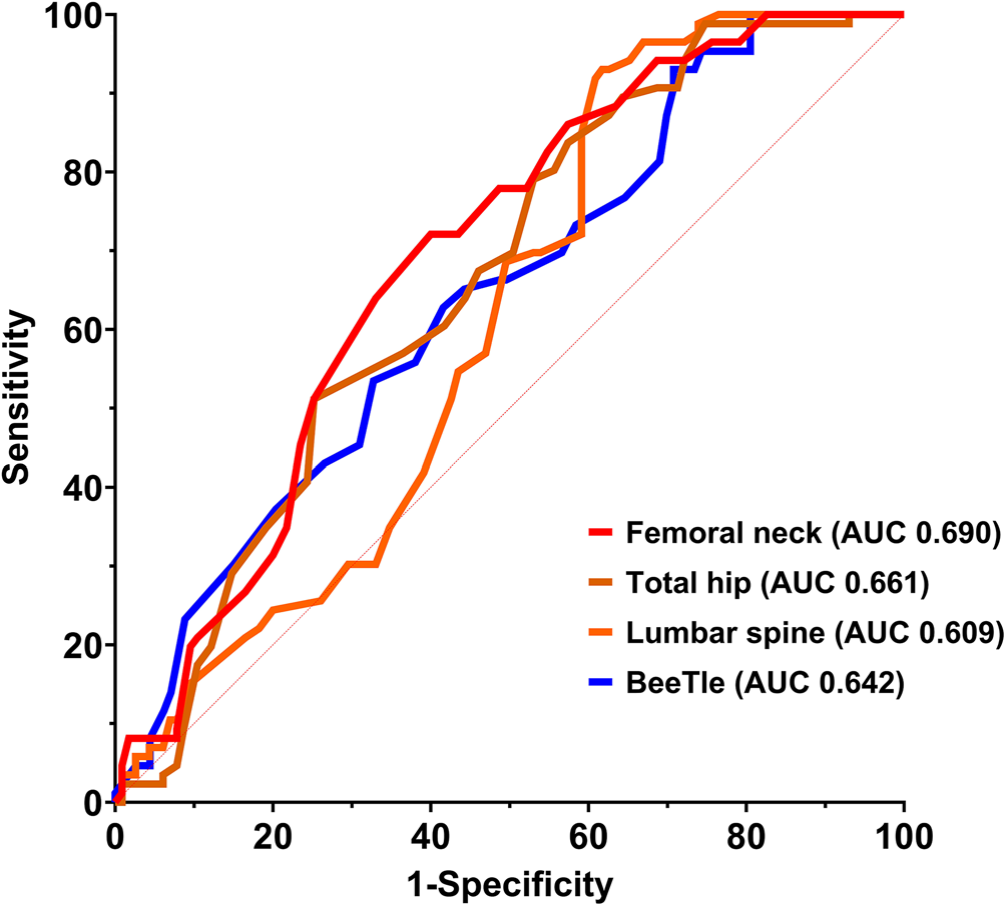
Receiver operating characteristics (ROC) curves and area under the curve (AUC) for Osteosys BeeTLE and DXA T-score in discriminating patients with or without prevalent fragility fractures.

We then stratified the study population in patients with prevalent fragility fractures and non-fractured patients. As expected, T-score levels were lower on average in patients with fracture compared to non-fractured patients (Fig. 4). Figure 5 shows the receiver operating characteristics (ROC) curves and area under the curve (AUC) for Osteosys BeeTLE and DXA T-score in discriminating fracture and non-fractured patients (AUC comparison was not significant).

The Osteosys BeeTLE instrument showed a coefficient of variation (CV, in 75 repeated measurements) of 1.21% with LSC at 95% CI of 3.40, which was not statistically different from the CV of DXA (1.20%).

## Discussion

Our study delves into the diagnostic accuracy of Osteosys BeeTLE, a point-of-care calcaneal ultrasound densitometer, in assessing BMD, offering potential advancements in osteoporosis management. Our findings reveal several critical insights into the role of Osteosys BeeTLE in osteoporosis diagnosis and screening.

One of the key findings from our study is the strong correlation observed between Osteosys BeeTLE and DXA measurements, as indicated by the association between BMD levels and BQI. These results underscore the potential of Osteosys BeeTLE to provide accurate BMD assessments, especially when considering the challenges associated with DXA, such as limited accessibility and portability. The establishment of a robust correlation between Osteosys BeeTLE and DXA suggests that Osteosys BeeTLE could be a valuable tool for diagnosing osteoporosis and assessing bone health in various clinical settings. In terms of diagnostic accuracy, our study revealed encouraging results. Osteosys BeeTLE demonstrated the ability to discriminate between “osteoporotic” and “non-osteoporotic” patients with noteworthy sensitivity and specificity when using DXA as the reference standard. While an AUC below 0.80 may suggest a lower level of accuracy compared to highly precise diagnostic tests, it still holds clinical relevance. In our context, this range indicates that Osteosys BeeTLE possesses some discriminatory power in distinguishing between fractured and non-fractured patients, although it may not be considered a highly accurate standalone diagnostic tool. Nonetheless, DXA yielded similar results. This is not surprising and has been previously reported that BMD alone should not be used for risk stratifying but should be corroborated by clinical risk factors. Yet, the clinical utility of such a test, particularly in initial risk assessment and screening scenarios, should not be underestimated as it can aid in identifying individuals who may benefit from further clinical and radiological evaluation.

The Cohen’s Kappa (K) values, which measure agreement beyond chance, indicate slight to fair agreement between Osteosys BeeTLE and DXA in classifying patients into diagnostic categories. This finding, together with the significant association between BMD and BQI, suggests that Osteosys BeeTLE T-score should be adjusted to the Italian population and further, large scale, studies are warranted. The Bland–Altman test, which assessed agreement and bias between Osteosys BeeTLE and DXA measurements, showed small differences, reaffirming Osteosys BeeTLE’s diagnostic accuracy. When considering the implications of our findings, it becomes evident that Osteosys BeeTLE holds promise as a complementary tool in osteoporosis management and screening. Its portability and accessibility make it suitable for a broader range of clinical settings, facilitating early diagnosis and intervention. While DXA remains invaluable, especially in specialized centers, Osteosys BeeTLE’s role in mass screening and primary care settings could enhance osteoporosis awareness and timely intervention.

To control for the potential impact of medications known to influence BMD, we conducted a sub-analysis specifically focusing on patients who had never been exposed to anti-osteoporosis medications. This sub-analysis aimed to assess diagnostic accuracy and agreement between Ostesys BeeTLE and DXA in a subset of patients more representative of the general, healthy, population. The results of this sub-analysis closely mirrored those of the overall analysis involving the complete dataset. The diagnostic accuracy, agreement, and correlations between Ostesys BeeTLE and DXA remained consistent, even in this subset of patients not influenced by anti-osteoporosis medications. These findings suggest that the performance of Ostesys BeeTLE in assessing BMD and bone quality remained robust across patient groups with varying medication histories. While the influence of medications on BMD is a well-recognized concern, our study suggests that Ostesys BeeTLE’s diagnostic accuracy holds steady, regardless of medication exposure, reinforcing its potential as a reliable tool for osteoporosis screening.

Other studies explored the efficacy and comparability of pulse-echo ultrasound with standard DXA. For example, van der Berg and colleagues published a study that has many similarities with ours^8^. The authors conducted a pilot study on 83 women with osteoporosis with both DXA and a pulse-echo ultrasound machine at the distal tibia. The AUC for detecting osteoporosis was remarkably similar to what we found in our analysis (femoral neck 0.813 vs. 0.812, total hip 0.720 vs. 0.784, lumbar spine 0.786 vs. 0.734). Again, the sensitivity and specificity of Osteosys BeeTLE was somehow comparable to other ultrasound instruments^9–11^. For example, Dovjak et al. recently reported a sensitivity of 94.4% and specificity of 59% for a similar ultrasound machine (we found a 90.2% sensitivity and 50.2% specificity vs DXA at lumbar spine)^11^.

Several limitations should be considered in interpreting our findings. First, the study population consisted of individuals of Caucasian ethnicity within a specific age range, which may limit the generalizability of our results to more diverse populations. Additionally, the study did not assess the impact of various comorbidities or medications on BMD measurements, which could influence diagnostic accuracy. Furthermore, the study focused on diagnostic accuracy and correlations, but additional research is needed to investigate the cost-effectiveness and feasibility of implementing Osteosys BeeTLE in various clinical settings. Lastly, while we considered DXA as the reference standard, it is not without its own limitations, which may have influenced our results. Moreover, we should aknowledge the inability of Ostesys BeeTLE to directly visualize vertebral fractures. This is a significant limitation in bone health assessment, as prevalent vertebral fractures are known to occur regardless of BMD T-scores. To address this limitation and enhance the comprehensiveness of osteoporosis diagnosis, supplementary imaging techniques such as Vertebral Fracture Assessment (VFA) are crucial. VFA, an advantage of DXA, provides valuable insights into vertebral fractures, contributing to a more holistic evaluation of bone health.

In conclusion, our study underscores the potential of Osteosys BeeTLE as a valuable adjunct to DXA in osteoporosis diagnosis and bone health assessment. While further validation and larger-scale studies are needed, the promising diagnostic accuracy, strong correlations, and minimal bias observed in our study support the integration of Osteosys BeeTLE into clinical practice. This innovative technology has the potential to address accessibility challenges associated with traditional DXA scans and contribute significantly to the early detection and management of osteoporosis on a broader scale.

### Supplementary Information

Supplementary Information.

## Data Availability

Data of the analysis are available upon reasonable request to Giovanni Adami (giovanni.adami@univr.it).

## Abbreviation

DXA: Dual-energy X-ray absorptiometry
