# Supplementary material for: New point-of-care calcaneal ultrasound densitometer (Osteosys BeeTLE) compared to standard dual-energy X-ray absorptiometry (DXA)
Source: Sci Rep. 2024 Mar 22;14:6898. doi: 10.1038/s41598-024-56787-8 (PMC10959987; doi:10.1038/s41598-024-56787-8)

**Supplementary materials**

**New Point-of-Care Calcaneal Ultrasound Densitometer (BeeTLe) compared to standard dual-energy X-ray absorptiometry (DXA**

**Authors:** Giovanni Adami^1^, Maurizio Rossini^1^, Davide Gatti^1^, Paolo Serpi^2^, Christian Fabrizio^2^, Roberto Lovato^3^

1. Rheumatology Unit, Azienda Ospedaliera Universitaria Integrata di Verona, Verona, Italy
2. Caresmed SRL, Milano, Italy
3. Bone Specialist Unit, Ospedale Casa di Cura Villa Berica, Vicenza, Italy

**Table of content**

**Table s1.** Sensitivity and specificity of BeeTLE BQI for detecting osteoporosis according to gold-standard DXA at femoral neck

**Table s2.** Sensitivity and specificity of BeeTLE BQI for detecting osteoporosis according to gold-standard DXA at total hip

**Table s3.** Sensitivity and specificity of BeeTLE BQI for detecting osteoporosis according to gold-standard DXA at lumbar spine

**Figure s1.** T-score distribution in the study population with Osteosys BeeTLE and standard DXA

**Figure s2.** ROC diagnostic accuracy of Osteosys Beetle in patients never exposed to anti-osteoporosis medications

**Figure s3.** Bland Altman graph for BeeTLe T scores and dual-energy X-ray absorptiometry (DXA) T scores in patients never exposed to anti-osteoporosis medications

**Table s1.** Sensitivity and specificity of BeeTLE BQI for detecting osteoporosis according to gold-standard DXA at femoral neck

| BeeTLE BQI | Sensitivity% | 95% CI | Specificity% | 95% CI | Likelihood ratio |
| --- | --- | --- | --- | --- | --- |
| < 37.20 | 1,852 | 0,09499% to 9,771% | 100,0 | 97,42% to 100,0% |  |
| < 40.40 | 3,704 | 0,6581% to 12,54% | 100,0 | 97,42% to 100,0% |  |
| < 41.05 | 3,704 | 0,6581% to 12,54% | 99,31 | 96,20% to 99,96% | 5,370 |
| < 41.80 | 7,407 | 2,918% to 17,55% | 99,31 | 96,20% to 99,96% | 10,74 |
| < 42.80 | 11,11 | 5,193% to 22,19% | 99,31 | 96,20% to 99,96% | 16,11 |
| < 43.95 | 14,81 | 7,703% to 26,60% | 99,31 | 96,20% to 99,96% | 21,48 |
| < 45.00 | 16,67 | 9,024% to 28,74% | 99,31 | 96,20% to 99,96% | 24,17 |
| < 45.70 | 18,52 | 10,38% to 30,84% | 99,31 | 96,20% to 99,96% | 26,85 |
| < 47.65 | 20,37 | 11,77% to 32,90% | 99,31 | 96,20% to 99,96% | 29,54 |
| < 49.45 | 24,07 | 14,64% to 36,95% | 99,31 | 96,20% to 99,96% | 34,91 |
| < 50.10 | 25,93 | 16,12% to 38,93% | 97,93 | 94,09% to 99,44% | 12,53 |
| < 50.75 | 27,78 | 17,62% to 40,89% | 97,93 | 94,09% to 99,44% | 13,43 |
| < 51.20 | 29,63 | 19,14% to 42,83% | 97,93 | 94,09% to 99,44% | 14,32 |
| < 51.80 | 31,48 | 20,68% to 44,74% | 97,93 | 94,09% to 99,44% | 15,22 |
| < 52.45 | 37,04 | 25,42% to 50,37% | 97,93 | 94,09% to 99,44% | 17,90 |
| < 52.85 | 37,04 | 25,42% to 50,37% | 97,24 | 93,12% to 98,92% | 13,43 |
| < 53.00 | 37,04 | 25,42% to 50,37% | 95,17 | 90,37% to 97,64% | 7,672 |
| < 53.20 | 38,89 | 27,04% to 52,21% | 95,17 | 90,37% to 97,64% | 8,056 |
| < 53.55 | 38,89 | 27,04% to 52,21% | 94,48 | 89,49% to 97,18% | 7,049 |
| < 54.05 | 40,74 | 28,68% to 54,03% | 93,79 | 88,63% to 96,70% | 6,564 |
| < 54.35 | 42,59 | 30,33% to 55,84% | 93,10 | 87,77% to 96,21% | 6,176 |
| < 54.50 | 42,59 | 30,33% to 55,84% | 91,72 | 86,09% to 95,20% | 5,147 |
| < 54.80 | 44,44 | 32,00% to 57,62% | 90,34 | 84,45% to 94,16% | 4,603 |
| < 55.15 | 44,44 | 32,00% to 57,62% | 89,66 | 83,63% to 93,63% | 4,296 |
| < 55.35 | 48,15 | 35,39% to 61,15% | 89,66 | 83,63% to 93,63% | 4,654 |
| < 55.50 | 50,00 | 37,11% to 62,89% | 89,66 | 83,63% to 93,63% | 4,833 |
| < 55.75 | 51,85 | 38,85% to 64,61% | 89,66 | 83,63% to 93,63% | 5,012 |
| < 56.00 | 51,85 | 38,85% to 64,61% | 88,97 | 82,83% to 93,09% | 4,699 |
| < 56.15 | 51,85 | 38,85% to 64,61% | 88,28 | 82,03% to 92,55% | 4,423 |
| < 56.35 | 51,85 | 38,85% to 64,61% | 86,21 | 79,65% to 90,89% | 3,759 |
| < 56.55 | 51,85 | 38,85% to 64,61% | 85,52 | 78,87% to 90,33% | 3,580 |
| < 56.80 | 53,70 | 40,61% to 66,31% | 85,52 | 78,87% to 90,33% | 3,708 |
| < 57.10 | 53,70 | 40,61% to 66,31% | 83,45 | 76,55% to 88,62% | 3,245 |
| < 57.40 | 53,70 | 40,61% to 66,31% | 82,07 | 75,02% to 87,46% | 2,995 |
| < 57.65 | 53,70 | 40,61% to 66,31% | 80,69 | 73,51% to 86,29% | 2,781 |
| < 57.75 | 55,56 | 42,38% to 68,00% | 80,69 | 73,51% to 86,29% | 2,877 |
| < 57.90 | 55,56 | 42,38% to 68,00% | 80,00 | 72,75% to 85,70% | 2,778 |
| < 58.25 | 55,56 | 42,38% to 68,00% | 76,55 | 69,03% to 82,71% | 2,369 |
| < 58.55 | 55,56 | 42,38% to 68,00% | 75,86 | 68,29% to 82,10% | 2,302 |
| < 58.70 | 55,56 | 42,38% to 68,00% | 75,17 | 67,55% to 81,49% | 2,238 |
| < 59.30 | 57,41 | 44,16% to 69,67% | 75,17 | 67,55% to 81,49% | 2,312 |
| < 59.90 | 57,41 | 44,16% to 69,67% | 74,48 | 66,82% to 80,88% | 2,250 |
| < 60.25 | 59,26 | 45,97% to 71,32% | 74,48 | 66,82% to 80,88% | 2,322 |
| < 60.70 | 62,96 | 49,63% to 74,58% | 73,79 | 66,09% to 80,27% | 2,403 |
| < 60.95 | 62,96 | 49,63% to 74,58% | 73,10 | 65,36% to 79,66% | 2,341 |
| < 61.25 | 62,96 | 49,63% to 74,58% | 72,41 | 64,63% to 79,04% | 2,282 |
| < 61.65 | 62,96 | 49,63% to 74,58% | 71,03 | 63,18% to 77,80% | 2,174 |
| < 61.85 | 62,96 | 49,63% to 74,58% | 69,66 | 61,74% to 76,55% | 2,075 |
| < 61.95 | 64,81 | 51,48% to 76,18% | 69,66 | 61,74% to 76,55% | 2,136 |
| < 62.15 | 68,52 | 55,26% to 79,32% | 69,66 | 61,74% to 76,55% | 2,258 |
| < 62.45 | 68,52 | 55,26% to 79,32% | 68,97 | 61,03% to 75,92% | 2,208 |
| < 62.85 | 68,52 | 55,26% to 79,32% | 68,28 | 60,31% to 75,30% | 2,160 |
| < 63.15 | 70,37 | 57,17% to 80,86% | 68,28 | 60,31% to 75,30% | 2,218 |
| < 63.70 | 72,22 | 59,11% to 82,38% | 68,28 | 60,31% to 75,30% | 2,277 |
| < 64.25 | 72,22 | 59,11% to 82,38% | 66,90 | 58,89% to 74,03% | 2,182 |
| **< 64.55** | **75,93** | **63,05% to 85,36%** | **66,90** | **58,89% to 74,03%** | **2,294** |
| < 64.85 | 75,93 | 63,05% to 85,36% | 65,52 | 57,47% to 72,76% | 2,202 |
| < 65.00 | 75,93 | 63,05% to 85,36% | 64,83 | 56,76% to 72,13% | 2,159 |
| < 65.45 | 75,93 | 63,05% to 85,36% | 62,76 | 54,66% to 70,20% | 2,039 |
| < 66.00 | 77,78 | 65,06% to 86,80% | 62,07 | 53,96% to 69,56% | 2,051 |
| < 66.25 | 77,78 | 65,06% to 86,80% | 61,38 | 53,26% to 68,91% | 2,014 |
| < 66.60 | 77,78 | 65,06% to 86,80% | 59,31 | 51,17% to 66,97% | 1,911 |
| < 67.15 | 77,78 | 65,06% to 86,80% | 58,62 | 50,48% to 66,31% | 1,880 |
| < 67.60 | 77,78 | 65,06% to 86,80% | 57,93 | 49,79% to 65,66% | 1,849 |
| < 67.85 | 77,78 | 65,06% to 86,80% | 56,55 | 48,42% to 64,35% | 1,790 |
| < 68.35 | 79,63 | 67,10% to 88,23% | 56,55 | 48,42% to 64,35% | 1,833 |
| < 69.20 | 79,63 | 67,10% to 88,23% | 55,17 | 47,05% to 63,03% | 1,776 |
| < 69.65 | 79,63 | 67,10% to 88,23% | 53,79 | 45,69% to 61,71% | 1,723 |
| < 69.80 | 79,63 | 67,10% to 88,23% | 53,10 | 45,01% to 61,04% | 1,698 |
| < 70.50 | 79,63 | 67,10% to 88,23% | 52,41 | 44,33% to 60,37% | 1,673 |
| < 71.80 | 79,63 | 67,10% to 88,23% | 51,72 | 43,65% to 59,71% | 1,649 |
| < 72.65 | 81,48 | 69,16% to 89,62% | 51,03 | 42,98% to 59,04% | 1,664 |
| < 73.30 | 81,48 | 69,16% to 89,62% | 50,34 | 42,30% to 58,37% | 1,641 |
| < 73.85 | 81,48 | 69,16% to 89,62% | 48,28 | 40,29% to 56,35% | 1,575 |
| < 74.05 | 81,48 | 69,16% to 89,62% | 46,21 | 38,29% to 54,31% | 1,515 |
| < 74.30 | 81,48 | 69,16% to 89,62% | 44,83 | 36,97% to 52,95% | 1,477 |
| < 74.50 | 81,48 | 69,16% to 89,62% | 44,14 | 36,31% to 52,27% | 1,459 |
| < 74.90 | 81,48 | 69,16% to 89,62% | 42,76 | 35,00% to 50,90% | 1,423 |
| < 75.65 | 81,48 | 69,16% to 89,62% | 42,07 | 34,34% to 50,21% | 1,407 |
| < 76.25 | 83,33 | 71,26% to 90,98% | 42,07 | 34,34% to 50,21% | 1,438 |
| < 76.50 | 83,33 | 71,26% to 90,98% | 40,00 | 32,38% to 48,13% | 1,389 |
| < 76.85 | 83,33 | 71,26% to 90,98% | 39,31 | 31,73% to 47,44% | 1,373 |
| < 77.25 | 83,33 | 71,26% to 90,98% | 38,62 | 31,09% to 46,74% | 1,358 |
| < 77.45 | 83,33 | 71,26% to 90,98% | 37,24 | 29,80% to 45,34% | 1,328 |
| < 77.55 | 83,33 | 71,26% to 90,98% | 36,55 | 29,15% to 44,64% | 1,313 |
| < 77.65 | 85,19 | 73,40% to 92,30% | 36,55 | 29,15% to 44,64% | 1,343 |
| < 77.90 | 85,19 | 73,40% to 92,30% | 35,86 | 28,51% to 43,94% | 1,328 |
| < 78.25 | 85,19 | 73,40% to 92,30% | 35,17 | 27,87% to 43,24% | 1,314 |
| < 78.55 | 85,19 | 73,40% to 92,30% | 34,48 | 27,24% to 42,53% | 1,300 |
| < 78.75 | 85,19 | 73,40% to 92,30% | 32,41 | 25,33% to 40,40% | 1,260 |
| < 79.10 | 90,74 | 80,09% to 95,98% | 32,41 | 25,33% to 40,40% | 1,343 |
| < 79.50 | 90,74 | 80,09% to 95,98% | 31,72 | 24,70% to 39,69% | 1,329 |
| < 79.90 | 90,74 | 80,09% to 95,98% | 31,03 | 24,08% to 38,97% | 1,316 |
| < 80.70 | 90,74 | 80,09% to 95,98% | 29,66 | 22,82% to 37,54% | 1,290 |
| < 81.95 | 96,30 | 87,46% to 99,34% | 28,97 | 22,20% to 36,82% | 1,356 |
| < 83.10 | 96,30 | 87,46% to 99,34% | 26,90 | 20,34% to 34,64% | 1,317 |
| < 84.20 | 96,30 | 87,46% to 99,34% | 26,21 | 19,73% to 33,91% | 1,305 |
| < 85.70 | 96,30 | 87,46% to 99,34% | 24,83 | 18,51% to 32,45% | 1,281 |
| < 88.40 | 96,30 | 87,46% to 99,34% | 22,76 | 16,69% to 30,23% | 1,247 |
| < 90.50 | 100,0 | 93,36% to 100,0% | 22,76 | 16,69% to 30,23% | 1,295 |
| < 91.50 | 100,0 | 93,36% to 100,0% | 22,07 | 16,09% to 29,49% | 1,283 |
| < 92.35 | 100,0 | 93,36% to 100,0% | 21,38 | 15,49% to 28,75% | 1,272 |
| < 93.00 | 100,0 | 93,36% to 100,0% | 19,31 | 13,71% to 26,49% | 1,239 |
| < 94.60 | 100,0 | 93,36% to 100,0% | 17,24 | 11,96% to 24,21% | 1,208 |
| < 95.65 | 100,0 | 93,36% to 100,0% | 15,17 | 10,24% to 21,90% | 1,179 |
| < 96.30 | 100,0 | 93,36% to 100,0% | 14,48 | 9,672% to 21,13% | 1,169 |
| < 97.75 | 100,0 | 93,36% to 100,0% | 12,41 | 7,998% to 18,77% | 1,142 |
| < 99.50 | 100,0 | 93,36% to 100,0% | 10,34 | 6,370% to 16,37% | 1,115 |
| < 102.6 | 100,0 | 93,36% to 100,0% | 8,276 | 4,797% to 13,91% | 1,090 |
| < 107.4 | 100,0 | 93,36% to 100,0% | 6,207 | 3,300% to 11,37% | 1,066 |
| < 111.8 | 100,0 | 93,36% to 100,0% | 5,517 | 2,822% to 10,51% | 1,058 |
| < 114.3 | 100,0 | 93,36% to 100,0% | 3,448 | 1,482% to 7,818% | 1,036 |
| < 125.3 | 100,0 | 93,36% to 100,0% | 2,069 | 0,5639% to 5,906% | 1,021 |

**Table s2.** Sensitivity and specificity of BeeTLE BQI for detecting osteoporosis according to gold-standard DXA at total hip

|  | Sensitivity% | 95% CI | Specificity% | 95% CI | Likelihood ratio |
| --- | --- | --- | --- | --- | --- |
| < 37.20 | 2,381 | 0,1221% to 12,32% | 100,0 | 97,61% to 100,0% |  |
| < 40.40 | 4,762 | 0,8461% to 15,79% | 100,0 | 97,61% to 100,0% |  |
| < 41.05 | 4,762 | 0,8461% to 15,79% | 99,36 | 96,48% to 99,97% | 7,476 |
| < 41.80 | 9,524 | 3,766% to 22,07% | 99,36 | 96,48% to 99,97% | 14,95 |
| < 42.80 | 9,524 | 3,766% to 22,07% | 98,09 | 94,53% to 99,48% | 4,984 |
| < 43.95 | 9,524 | 3,766% to 22,07% | 96,82 | 92,76% to 98,63% | 2,990 |
| < 45.00 | 9,524 | 3,766% to 22,07% | 96,18 | 91,91% to 98,24% | 2,492 |
| < 45.70 | 11,90 | 5,194% to 25,00% | 96,18 | 91,91% to 98,24% | 3,115 |
| < 47.65 | 14,29 | 6,716% to 27,84% | 96,18 | 91,91% to 98,24% | 3,738 |
| < 49.45 | 14,29 | 6,716% to 27,84% | 94,90 | 90,27% to 97,40% | 2,804 |
| < 50.10 | 16,67 | 8,316% to 30,60% | 93,63 | 88,67% to 96,50% | 2,617 |
| < 50.75 | 19,05 | 9,982% to 33,30% | 93,63 | 88,67% to 96,50% | 2,990 |
| < 51.20 | 19,05 | 9,982% to 33,30% | 92,99 | 87,89% to 96,04% | 2,719 |
| < 51.80 | 21,43 | 11,71% to 35,94% | 92,99 | 87,89% to 96,04% | 3,058 |
| < 52.45 | 28,57 | 17,17% to 43,57% | 92,99 | 87,89% to 96,04% | 4,078 |
| < 52.85 | 28,57 | 17,17% to 43,57% | 92,36 | 87,12% to 95,57% | 3,738 |
| < 53.00 | 35,71 | 22,99% to 50,83% | 92,36 | 87,12% to 95,57% | 4,673 |
| < 53.20 | 35,71 | 22,99% to 50,83% | 91,72 | 86,35% to 95,10% | 4,313 |
| < 53.55 | 35,71 | 22,99% to 50,83% | 91,08 | 85,59% to 94,61% | 4,005 |
| < 54.05 | 38,10 | 25,00% to 53,19% | 90,45 | 84,84% to 94,12% | 3,987 |
| < 54.35 | 40,48 | 27,04% to 55,51% | 89,81 | 84,09% to 93,63% | 3,972 |
| < 54.50 | 40,48 | 27,04% to 55,51% | 88,54 | 82,61% to 92,62% | 3,530 |
| < 54.80 | 45,24 | 31,22% to 60,05% | 87,90 | 81,87% to 92,11% | 3,738 |
| < 55.15 | 45,24 | 31,22% to 60,05% | 87,26 | 81,14% to 91,60% | 3,551 |
| < 55.35 | 45,24 | 31,22% to 60,05% | 85,99 | 79,69% to 90,56% | 3,228 |
| < 55.50 | 45,24 | 31,22% to 60,05% | 85,35 | 78,98% to 90,04% | 3,088 |
| < 55.75 | 45,24 | 31,22% to 60,05% | 84,71 | 78,26% to 89,51% | 2,959 |
| < 56.00 | 45,24 | 31,22% to 60,05% | 84,08 | 77,55% to 88,98% | 2,841 |
| < 56.15 | 47,62 | 33,36% to 62,28% | 84,08 | 77,55% to 88,98% | 2,990 |
| < 56.35 | 50,00 | 35,53% to 64,47% | 82,80 | 76,13% to 87,90% | 2,907 |
| < 56.55 | 50,00 | 35,53% to 64,47% | 82,17 | 75,43% to 87,36% | 2,804 |
| < 56.80 | 50,00 | 35,53% to 64,47% | 81,53 | 74,73% to 86,82% | 2,707 |
| < 57.10 | 50,00 | 35,53% to 64,47% | 79,62 | 72,64% to 85,18% | 2,453 |
| < 57.40 | 54,76 | 39,95% to 68,78% | 79,62 | 72,64% to 85,18% | 2,687 |
| < 57.65 | 54,76 | 39,95% to 68,78% | 78,34 | 71,27% to 84,07% | 2,529 |
| < 57.75 | 57,14 | 42,21% to 70,88% | 78,34 | 71,27% to 84,07% | 2,639 |
| < 57.90 | 57,14 | 42,21% to 70,88% | 77,71 | 70,58% to 83,51% | 2,563 |
| < 58.25 | 57,14 | 42,21% to 70,88% | 74,52 | 67,18% to 80,70% | 2,243 |
| < 58.55 | 57,14 | 42,21% to 70,88% | 73,89 | 66,50% to 80,13% | 2,188 |
| < 58.70 | 57,14 | 42,21% to 70,88% | 73,25 | 65,83% to 79,56% | 2,136 |
| < 59.30 | 57,14 | 42,21% to 70,88% | 72,61 | 65,16% to 78,98% | 2,086 |
| < 59.90 | 57,14 | 42,21% to 70,88% | 71,97 | 64,49% to 78,41% | 2,039 |
| < 60.25 | 59,52 | 44,49% to 72,96% | 71,97 | 64,49% to 78,41% | 2,124 |
| < 60.70 | 61,90 | 46,81% to 75,00% | 70,70 | 63,16% to 77,26% | 2,113 |
| < 60.95 | 61,90 | 46,81% to 75,00% | 70,06 | 62,49% to 76,68% | 2,068 |
| < 61.25 | 61,90 | 46,81% to 75,00% | 69,43 | 61,83% to 76,10% | 2,025 |
| < 61.65 | 61,90 | 46,81% to 75,00% | 68,15 | 60,51% to 74,93% | 1,944 |
| < 61.85 | 61,90 | 46,81% to 75,00% | 66,88 | 59,19% to 73,76% | 1,869 |
| < 61.95 | 61,90 | 46,81% to 75,00% | 66,24 | 58,54% to 73,17% | 1,834 |
| < 62.15 | 66,67 | 51,55% to 78,99% | 66,24 | 58,54% to 73,17% | 1,975 |
| < 62.45 | 66,67 | 51,55% to 78,99% | 65,61 | 57,88% to 72,58% | 1,938 |
| < 62.85 | 69,05 | 53,97% to 80,93% | 65,61 | 57,88% to 72,58% | 2,007 |
| < 63.15 | 71,43 | 56,43% to 82,83% | 65,61 | 57,88% to 72,58% | 2,077 |
| **< 63.70** | **73,81** | **58,93% to 84,70%** | **65,61** | **57,88% to 72,58%** | **2,146** |
| < 64.25 | 73,81 | 58,93% to 84,70% | 64,33 | 56,58% to 71,40% | 2,069 |
| < 64.55 | 73,81 | 58,93% to 84,70% | 63,06 | 55,28% to 70,21% | 1,998 |
| < 64.85 | 73,81 | 58,93% to 84,70% | 61,78 | 53,99% to 69,02% | 1,931 |
| < 65.00 | 73,81 | 58,93% to 84,70% | 61,15 | 53,34% to 68,42% | 1,900 |
| < 65.45 | 73,81 | 58,93% to 84,70% | 59,24 | 51,42% to 66,61% | 1,811 |
| < 66.00 | 78,57 | 64,06% to 88,29% | 59,24 | 51,42% to 66,61% | 1,927 |
| < 66.25 | 78,57 | 64,06% to 88,29% | 58,60 | 50,78% to 66,01% | 1,898 |
| < 66.60 | 78,57 | 64,06% to 88,29% | 56,69 | 48,87% to 64,19% | 1,814 |
| < 67.15 | 78,57 | 64,06% to 88,29% | 56,05 | 48,23% to 63,58% | 1,788 |
| < 67.60 | 78,57 | 64,06% to 88,29% | 55,41 | 47,60% to 62,97% | 1,762 |
| < 67.85 | 78,57 | 64,06% to 88,29% | 54,14 | 46,34% to 61,74% | 1,713 |
| < 68.35 | 80,95 | 66,70% to 90,02% | 54,14 | 46,34% to 61,74% | 1,765 |
| < 69.20 | 80,95 | 66,70% to 90,02% | 52,87 | 45,08% to 60,51% | 1,718 |
| < 69.65 | 80,95 | 66,70% to 90,02% | 51,59 | 43,83% to 59,28% | 1,672 |
| < 69.80 | 80,95 | 66,70% to 90,02% | 50,96 | 43,21% to 58,66% | 1,651 |
| < 70.50 | 80,95 | 66,70% to 90,02% | 50,32 | 42,58% to 58,04% | 1,629 |
| < 71.80 | 80,95 | 66,70% to 90,02% | 49,68 | 41,96% to 57,42% | 1,609 |
| < 72.65 | 80,95 | 66,70% to 90,02% | 48,41 | 40,72% to 56,17% | 1,569 |
| < 73.30 | 80,95 | 66,70% to 90,02% | 47,77 | 40,10% to 55,54% | 1,550 |
| < 73.85 | 80,95 | 66,70% to 90,02% | 45,86 | 38,26% to 53,66% | 1,495 |
| < 74.05 | 88,10 | 75,00% to 94,81% | 45,86 | 38,26% to 53,66% | 1,627 |
| < 74.30 | 88,10 | 75,00% to 94,81% | 44,59 | 37,03% to 52,40% | 1,590 |
| < 74.50 | 88,10 | 75,00% to 94,81% | 43,95 | 36,42% to 51,77% | 1,572 |
| < 74.90 | 92,86 | 80,99% to 97,54% | 43,95 | 36,42% to 51,77% | 1,657 |
| < 75.65 | 92,86 | 80,99% to 97,54% | 43,31 | 35,81% to 51,13% | 1,638 |
| < 76.25 | 92,86 | 80,99% to 97,54% | 42,68 | 35,20% to 50,50% | 1,620 |
| < 76.50 | 92,86 | 80,99% to 97,54% | 40,76 | 33,39% to 48,58% | 1,568 |
| < 76.85 | 92,86 | 80,99% to 97,54% | 40,13 | 32,78% to 47,94% | 1,551 |
| < 77.25 | 92,86 | 80,99% to 97,54% | 39,49 | 32,18% to 47,30% | 1,535 |
| < 77.45 | 92,86 | 80,99% to 97,54% | 38,22 | 30,98% to 46,01% | 1,503 |
| < 77.55 | 92,86 | 80,99% to 97,54% | 37,58 | 30,39% to 45,37% | 1,488 |
| < 77.65 | 92,86 | 80,99% to 97,54% | 36,94 | 29,79% to 44,72% | 1,473 |
| < 77.90 | 92,86 | 80,99% to 97,54% | 36,31 | 29,19% to 44,07% | 1,458 |
| < 78.25 | 92,86 | 80,99% to 97,54% | 35,67 | 28,60% to 43,42% | 1,443 |
| < 78.55 | 92,86 | 80,99% to 97,54% | 35,03 | 28,01% to 42,77% | 1,429 |
| < 78.75 | 92,86 | 80,99% to 97,54% | 33,12 | 26,24% to 40,81% | 1,388 |
| < 79.10 | 100,0 | 91,62% to 100,0% | 33,12 | 26,24% to 40,81% | 1,495 |
| < 79.50 | 100,0 | 91,62% to 100,0% | 32,48 | 25,65% to 40,15% | 1,481 |
| < 79.90 | 100,0 | 91,62% to 100,0% | 31,85 | 25,07% to 39,49% | 1,467 |
| < 80.70 | 100,0 | 91,62% to 100,0% | 30,57 | 23,90% to 38,17% | 1,440 |
| < 81.95 | 100,0 | 91,62% to 100,0% | 28,03 | 21,59% to 35,51% | 1,389 |
| < 83.10 | 100,0 | 91,62% to 100,0% | 26,11 | 19,87% to 33,50% | 1,353 |
| < 84.20 | 100,0 | 91,62% to 100,0% | 25,48 | 19,30% to 32,82% | 1,342 |
| < 85.70 | 100,0 | 91,62% to 100,0% | 24,20 | 18,17% to 31,47% | 1,319 |
| < 88.40 | 100,0 | 91,62% to 100,0% | 22,29 | 16,49% to 29,42% | 1,287 |
| < 90.50 | 100,0 | 91,62% to 100,0% | 21,02 | 15,38% to 28,05% | 1,266 |
| < 91.50 | 100,0 | 91,62% to 100,0% | 20,38 | 14,82% to 27,36% | 1,256 |
| < 92.35 | 100,0 | 91,62% to 100,0% | 19,75 | 14,27% to 26,66% | 1,246 |
| < 93.00 | 100,0 | 91,62% to 100,0% | 17,83 | 12,64% to 24,57% | 1,217 |
| < 94.60 | 100,0 | 91,62% to 100,0% | 15,92 | 11,02% to 22,45% | 1,189 |
| < 95.65 | 100,0 | 91,62% to 100,0% | 14,01 | 9,439% to 20,31% | 1,163 |
| < 96.30 | 100,0 | 91,62% to 100,0% | 13,38 | 8,918% to 19,58% | 1,154 |
| < 97.75 | 100,0 | 91,62% to 100,0% | 11,46 | 7,376% to 17,39% | 1,129 |
| < 99.50 | 100,0 | 91,62% to 100,0% | 9,554 | 5,876% to 15,16% | 1,106 |
| < 102.6 | 100,0 | 91,62% to 100,0% | 7,643 | 4,426% to 12,88% | 1,083 |
| < 107.4 | 100,0 | 91,62% to 100,0% | 5,732 | 3,045% to 10,53% | 1,061 |
| < 111.8 | 100,0 | 91,62% to 100,0% | 5,096 | 2,604% to 9,732% | 1,054 |
| < 114.3 | 100,0 | 91,62% to 100,0% | 3,185 | 1,368% to 7,238% | 1,033 |
| < 125.3 | 100,0 | 91,62% to 100,0% | 1,911 | 0,5208% to 5,467% | 1,019 |

**Table s3.** Sensitivity and specificity of BeeTLE BQI for detecting osteoporosis according to gold-standard DXA at lumbar spine

|  | Sensitivity% | 95% CI | Specificity% | 95% CI | Likelihood ratio |
| --- | --- | --- | --- | --- | --- |
| < 37.20 | 0,9804 | 0,05029% to 5,346% | 100,0 | 96,19% to 100,0% |  |
| < 40.40 | 1,961 | 0,3484% to 6,869% | 100,0 | 96,19% to 100,0% |  |
| < 41.05 | 1,961 | 0,3484% to 6,869% | 98,97 | 94,39% to 99,95% | 1,902 |
| < 41.80 | 3,922 | 1,535% to 9,653% | 98,97 | 94,39% to 99,95% | 3,804 |
| < 42.80 | 5,882 | 2,724% to 12,24% | 98,97 | 94,39% to 99,95% | 5,706 |
| < 43.95 | 7,843 | 4,028% to 14,72% | 98,97 | 94,39% to 99,95% | 7,608 |
| < 45.00 | 8,824 | 4,712% to 15,92% | 98,97 | 94,39% to 99,95% | 8,559 |
| < 45.70 | 8,824 | 4,712% to 15,92% | 97,94 | 92,79% to 99,63% | 4,279 |
| < 47.65 | 9,804 | 5,413% to 17,11% | 97,94 | 92,79% to 99,63% | 4,755 |
| < 49.45 | 11,76 | 6,859% to 19,45% | 97,94 | 92,79% to 99,63% | 5,706 |
| < 50.10 | 13,73 | 8,355% to 21,73% | 96,91 | 91,30% to 99,16% | 4,438 |
| < 50.75 | 14,71 | 9,119% to 22,85% | 96,91 | 91,30% to 99,16% | 4,755 |
| < 51.20 | 15,69 | 9,892% to 23,97% | 96,91 | 91,30% to 99,16% | 5,072 |
| < 51.80 | 16,67 | 10,67% to 25,08% | 96,91 | 91,30% to 99,16% | 5,389 |
| < 52.45 | 19,61 | 13,07% to 28,35% | 96,91 | 91,30% to 99,16% | 6,340 |
| < 52.85 | 20,59 | 13,88% to 29,43% | 96,91 | 91,30% to 99,16% | 6,657 |
| < 53.00 | 23,53 | 16,35% to 32,63% | 96,91 | 91,30% to 99,16% | 7,608 |
| < 53.20 | 23,53 | 16,35% to 32,63% | 95,88 | 89,87% to 98,38% | 5,706 |
| < 53.55 | 23,53 | 16,35% to 32,63% | 94,85 | 88,50% to 97,78% | 4,565 |
| < 54.05 | 24,51 | 17,19% to 33,68% | 93,81 | 87,16% to 97,13% | 3,962 |
| < 54.35 | 24,51 | 17,19% to 33,68% | 91,75 | 84,56% to 95,76% | 2,972 |
| < 54.50 | 24,51 | 17,19% to 33,68% | 89,69 | 82,05% to 94,30% | 2,377 |
| < 54.80 | 27,45 | 19,73% to 36,81% | 89,69 | 82,05% to 94,30% | 2,663 |
| < 55.15 | 28,43 | 20,58% to 37,84% | 89,69 | 82,05% to 94,30% | 2,758 |
| < 55.35 | 28,43 | 20,58% to 37,84% | 87,63 | 79,61% to 92,78% | 2,298 |
| < 55.50 | 28,43 | 20,58% to 37,84% | 86,60 | 78,41% to 92,00% | 2,121 |
| < 55.75 | 29,41 | 21,45% to 38,87% | 86,60 | 78,41% to 92,00% | 2,195 |
| < 56.00 | 30,39 | 22,31% to 39,90% | 86,60 | 78,41% to 92,00% | 2,268 |
| < 56.15 | 30,39 | 22,31% to 39,90% | 85,57 | 77,22% to 91,20% | 2,106 |
| < 56.35 | 33,33 | 24,94% to 42,94% | 85,57 | 77,22% to 91,20% | 2,310 |
| < 56.55 | 34,31 | 25,82% to 43,95% | 85,57 | 77,22% to 91,20% | 2,377 |
| < 56.80 | 35,29 | 26,71% to 44,95% | 85,57 | 77,22% to 91,20% | 2,445 |
| < 57.10 | 35,29 | 26,71% to 44,95% | 82,47 | 73,71% to 88,76% | 2,014 |
| < 57.40 | 37,25 | 28,49% to 46,94% | 82,47 | 73,71% to 88,76% | 2,126 |
| < 57.65 | 39,22 | 30,30% to 48,92% | 82,47 | 73,71% to 88,76% | 2,238 |
| < 57.75 | 40,20 | 31,20% to 49,90% | 82,47 | 73,71% to 88,76% | 2,294 |
| < 57.90 | 40,20 | 31,20% to 49,90% | 81,44 | 72,56% to 87,93% | 2,166 |
| < 58.25 | 42,16 | 33,03% to 51,85% | 78,35 | 69,16% to 85,38% | 1,947 |
| < 58.55 | 42,16 | 33,03% to 51,85% | 77,32 | 68,04% to 84,52% | 1,859 |
| < 58.70 | 42,16 | 33,03% to 51,85% | 76,29 | 66,93% to 83,65% | 1,778 |
| < 59.30 | 43,14 | 33,95% to 52,83% | 76,29 | 66,93% to 83,65% | 1,819 |
| < 59.90 | 43,14 | 33,95% to 52,83% | 75,26 | 65,82% to 82,77% | 1,743 |
| < 60.25 | 44,12 | 34,87% to 53,79% | 75,26 | 65,82% to 82,77% | 1,783 |
| < 60.70 | 47,06 | 37,66% to 56,68% | 75,26 | 65,82% to 82,77% | 1,902 |
| < 60.95 | 48,04 | 38,59% to 57,63% | 75,26 | 65,82% to 82,77% | 1,942 |
| < 61.25 | 48,04 | 38,59% to 57,63% | 74,23 | 64,72% to 81,89% | 1,864 |
| < 61.65 | 50,00 | 40,47% to 59,53% | 74,23 | 64,72% to 81,89% | 1,940 |
| < 61.85 | 50,00 | 40,47% to 59,53% | 72,16 | 62,53% to 80,11% | 1,796 |
| < 61.95 | 50,98 | 41,42% to 60,47% | 72,16 | 62,53% to 80,11% | 1,832 |
| < 62.15 | 52,94 | 43,32% to 62,34% | 72,16 | 62,53% to 80,11% | 1,902 |
| < 62.45 | 53,92 | 44,28% to 63,28% | 72,16 | 62,53% to 80,11% | 1,937 |
| < 62.85 | 54,90 | 45,24% to 64,21% | 72,16 | 62,53% to 80,11% | 1,972 |
| < 63.15 | 55,88 | 46,21% to 65,13% | 72,16 | 62,53% to 80,11% | 2,008 |
| < 63.70 | 56,86 | 47,17% to 66,05% | 72,16 | 62,53% to 80,11% | 2,043 |
| < 64.25 | 58,82 | 49,12% to 67,88% | 72,16 | 62,53% to 80,11% | 2,113 |
| < 64.55 | 60,78 | 51,08% to 69,70% | 72,16 | 62,53% to 80,11% | 2,184 |
| < 64.85 | 62,75 | 53,06% to 71,51% | 72,16 | 62,53% to 80,11% | 2,254 |
| < 65.00 | 63,73 | 54,05% to 72,40% | 72,16 | 62,53% to 80,11% | 2,289 |
| < 65.45 | 63,73 | 54,05% to 72,40% | 69,07 | 59,30% to 77,40% | 2,060 |
| < 66.00 | 64,71 | 55,05% to 73,29% | 68,04 | 58,23% to 76,48% | 2,025 |
| < 66.25 | 65,69 | 56,05% to 74,18% | 68,04 | 58,23% to 76,48% | 2,055 |
| < 66.60 | 67,65 | 58,07% to 75,94% | 67,01 | 57,16% to 75,56% | 2,051 |
| < 67.15 | 67,65 | 58,07% to 75,94% | 65,98 | 56,10% to 74,64% | 1,988 |
| < 67.60 | 68,63 | 59,09% to 76,82% | 65,98 | 56,10% to 74,64% | 2,017 |
| < 67.85 | 68,63 | 59,09% to 76,82% | 63,92 | 54,00% to 72,78% | 1,902 |
| < 68.35 | 69,61 | 60,10% to 77,69% | 63,92 | 54,00% to 72,78% | 1,929 |
| < 69.20 | 69,61 | 60,10% to 77,69% | 61,86 | 51,91% to 70,90% | 1,825 |
| < 69.65 | 71,57 | 62,16% to 79,42% | 61,86 | 51,91% to 70,90% | 1,876 |
| < 69.80 | 72,55 | 63,19% to 80,27% | 61,86 | 51,91% to 70,90% | 1,902 |
| < 70.50 | 73,53 | 64,23% to 81,12% | 61,86 | 51,91% to 70,90% | 1,928 |
| < 71.80 | 74,51 | 65,27% to 81,97% | 61,86 | 51,91% to 70,90% | 1,953 |
| < 72.65 | 75,49 | 66,32% to 82,81% | 60,82 | 50,88% to 69,95% | 1,927 |
| < 73.30 | 76,47 | 67,37% to 83,65% | 60,82 | 50,88% to 69,95% | 1,952 |
| < 73.85 | 76,47 | 67,37% to 83,65% | 57,73 | 47,79% to 67,08% | 1,809 |
| < 74.05 | 79,41 | 70,57% to 86,12% | 57,73 | 47,79% to 67,08% | 1,879 |
| < 74.30 | 81,37 | 72,73% to 87,74% | 57,73 | 47,79% to 67,08% | 1,925 |
| < 74.50 | 82,35 | 73,82% to 88,54% | 57,73 | 47,79% to 67,08% | 1,948 |
| < 74.90 | 82,35 | 73,82% to 88,54% | 55,67 | 45,76% to 65,15% | 1,858 |
| < 75.65 | 83,33 | 74,92% to 89,33% | 55,67 | 45,76% to 65,15% | 1,880 |
| < 76.25 | 83,33 | 74,92% to 89,33% | 54,64 | 44,74% to 64,18% | 1,837 |
| < 76.50 | 85,29 | 77,15% to 90,88% | 53,61 | 43,74% to 63,21% | 1,839 |
| < 76.85 | 86,27 | 78,27% to 91,64% | 53,61 | 43,74% to 63,21% | 1,860 |
| < 77.25 | 86,27 | 78,27% to 91,64% | 52,58 | 42,73% to 62,23% | 1,819 |
| < 77.45 | 86,27 | 78,27% to 91,64% | 50,52 | 40,74% to 60,25% | 1,743 |
| < 77.55 | 87,25 | 79,41% to 92,40% | 50,52 | 40,74% to 60,25% | 1,763 |
| < 77.65 | 88,24 | 80,55% to 93,14% | 50,52 | 40,74% to 60,25% | 1,783 |
| < 77.90 | 89,22 | 81,71% to 93,87% | 50,52 | 40,74% to 60,25% | 1,803 |
| **< 78.25** | **90,20** | **82,89% to 94,59%** | **50,52** | **40,74% to 60,25%** | **1,823** |
| < 78.55 | 90,20 | 82,89% to 94,59% | 49,48 | 39,75% to 59,26% | 1,786 |
| < 78.75 | 90,20 | 82,89% to 94,59% | 46,39 | 36,79% to 56,26% | 1,683 |
| < 79.10 | 90,20 | 82,89% to 94,59% | 43,30 | 33,88% to 53,23% | 1,591 |
| < 79.50 | 90,20 | 82,89% to 94,59% | 42,27 | 32,92% to 52,21% | 1,562 |
| < 79.90 | 91,18 | 84,08% to 95,29% | 42,27 | 32,92% to 52,21% | 1,579 |
| < 80.70 | 93,14 | 86,51% to 96,64% | 42,27 | 32,92% to 52,21% | 1,613 |
| < 81.95 | 94,12 | 87,76% to 97,28% | 39,18 | 30,05% to 49,12% | 1,547 |
| < 83.10 | 94,12 | 87,76% to 97,28% | 36,08 | 27,22% to 46,00% | 1,472 |
| < 84.20 | 95,10 | 89,03% to 97,89% | 36,08 | 27,22% to 46,00% | 1,488 |
| < 85.70 | 95,10 | 89,03% to 97,89% | 34,02 | 25,36% to 43,90% | 1,441 |
| < 88.40 | 95,10 | 89,03% to 97,89% | 30,93 | 22,60% to 40,70% | 1,377 |
| < 90.50 | 97,06 | 91,71% to 99,20% | 30,93 | 22,60% to 40,70% | 1,405 |
| < 91.50 | 98,04 | 93,13% to 99,65% | 30,93 | 22,60% to 40,70% | 1,419 |
| < 92.35 | 99,02 | 94,65% to 99,95% | 30,93 | 22,60% to 40,70% | 1,434 |
| < 93.00 | 99,02 | 94,65% to 99,95% | 27,84 | 19,89% to 37,47% | 1,372 |
| < 94.60 | 99,02 | 94,65% to 99,95% | 24,74 | 17,23% to 34,18% | 1,316 |
| < 95.65 | 99,02 | 94,65% to 99,95% | 21,65 | 14,62% to 30,84% | 1,264 |
| < 96.30 | 100,0 | 96,37% to 100,0% | 21,65 | 14,62% to 30,84% | 1,276 |
| < 97.75 | 100,0 | 96,37% to 100,0% | 18,56 | 12,07% to 27,44% | 1,228 |
| < 99.50 | 100,0 | 96,37% to 100,0% | 15,46 | 9,601% to 23,96% | 1,183 |
| < 102.6 | 100,0 | 96,37% to 100,0% | 12,37 | 7,220% to 20,39% | 1,141 |
| < 107.4 | 100,0 | 96,37% to 100,0% | 9,278 | 4,958% to 16,70% | 1,102 |
| < 111.8 | 100,0 | 96,37% to 100,0% | 8,247 | 4,238% to 15,44% | 1,090 |
| < 114.3 | 100,0 | 96,37% to 100,0% | 5,155 | 2,222% to 11,50% | 1,054 |
| < 125.3 | 100,0 | 96,37% to 100,0% | 3,093 | 0,8430% to 8,702% | 1,032 |

**Figure s1.** T-score distribution in the study population with Osteosys BeeTLE and standard DXA


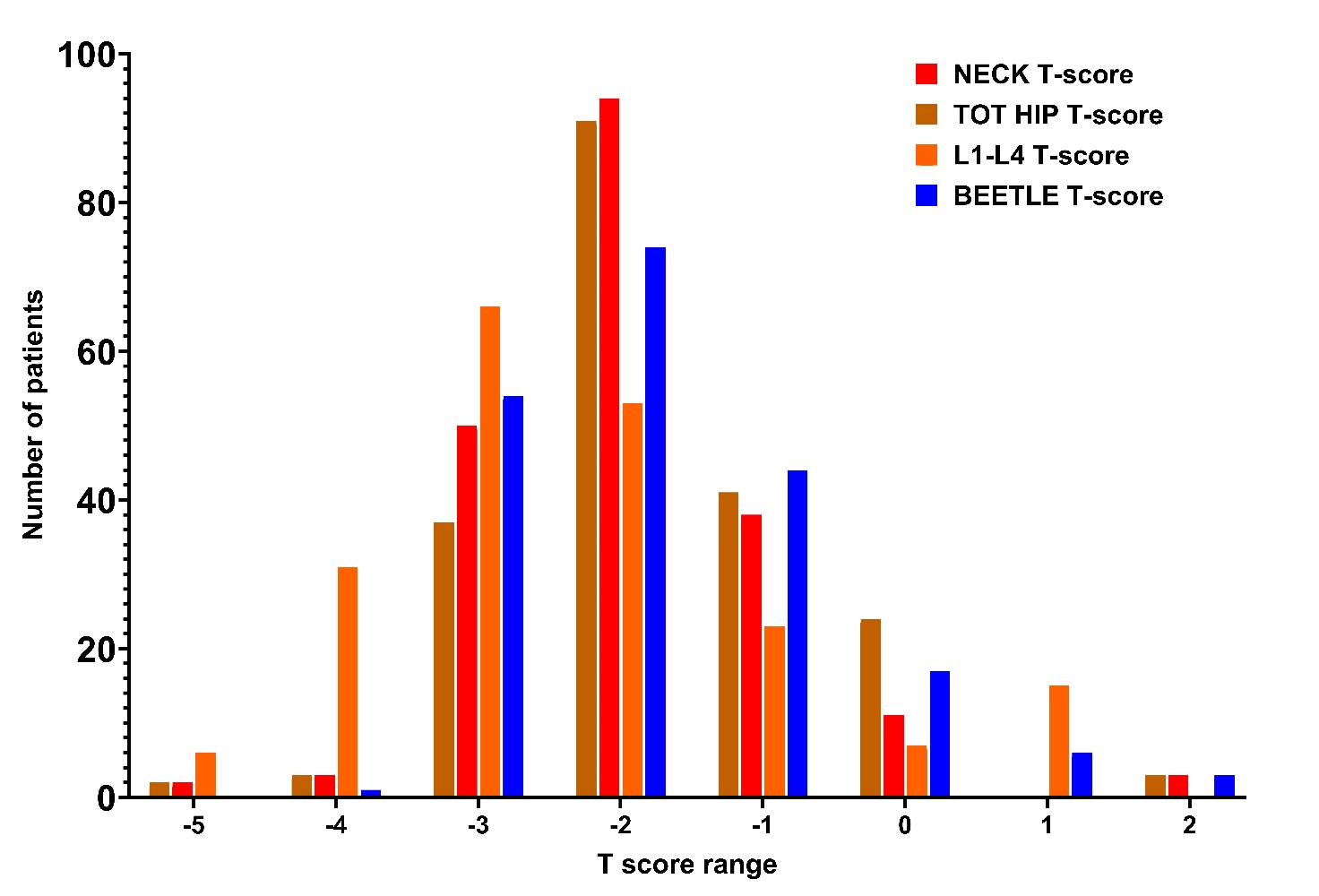


**Figure s2.** ROC diagnostic accuracy of Osteosys Beetle in patients never exposed to anti-osteoporosis medications


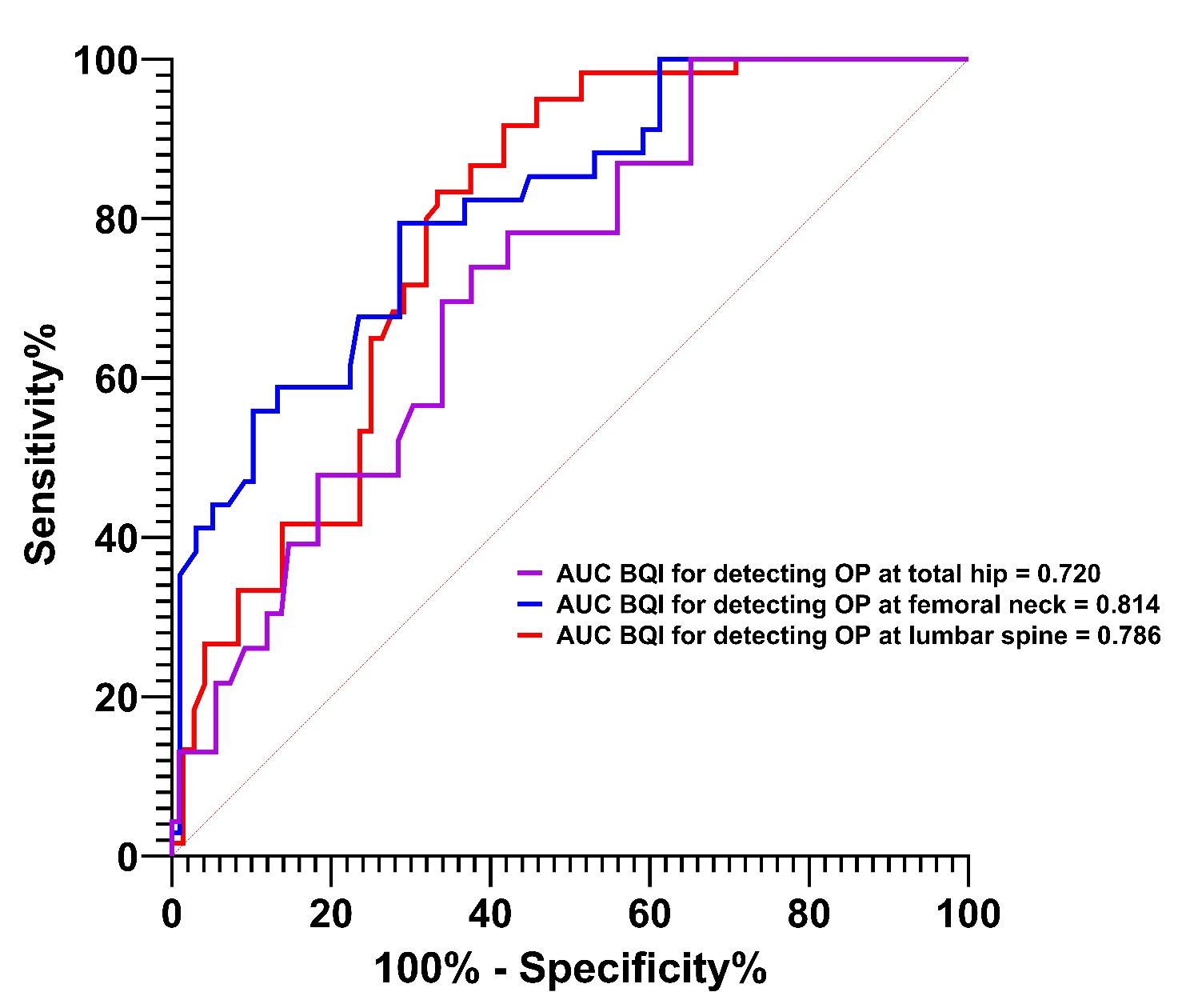


**Figure s3.** Bland Altman graph for BeeTLe T scores and dual-energy X-ray absorptiometry (DXA) T scores in patients never exposed to anti-osteoporosis medications


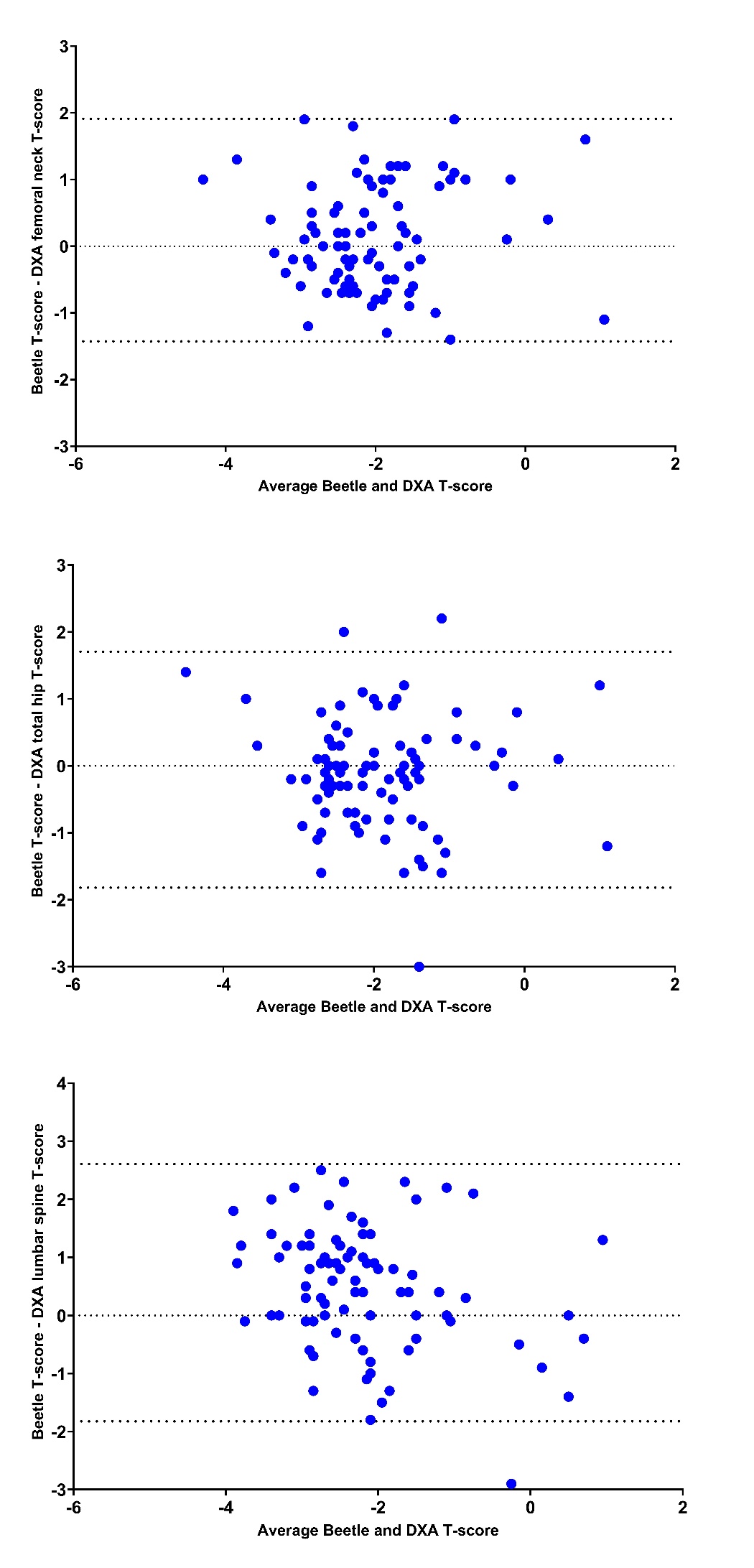

Supplement: Supplementary file 1 — Supplementary Information. [file 41598_2024_56787_MOESM1_ESM.docx]
